# Incidence of dislocation and associated risk factors in patients with a femoral neck fracture operated with an uncemented hemiarthroplasty

**DOI:** 10.1186/s12891-024-07237-z

**Published:** 2024-02-09

**Authors:** Britt Aaen Olesen, Susanne Faurholt Närhi, Thomas Giver Jensen, Søren Overgaard, Henrik Palm, Michala Skovlund Sørensen

**Affiliations:** 1 Department of Orthopaedic Surgery and Traumatology, Copenhagen University Hospital, Bispebjerg and Frederiksberg, Nielsine Nielsens Vej 5, Staircase 6, 2. Floor, Copenhagen, NV 2400 Denmark; 2 Department of Clinical Medicine, Faculty of Health and Medical Sciences, University of Copenhagen, Blegdamsvej 3B, 2200 Copenhagen N, Denmark

**Keywords:** Femoral neck fracture, Uncemented hip hemiarthroplasty, Dislocation, Incidence, Survival probability, Risk factors, Dementia, Posterior surgical approach

## Abstract

**Background:**

Several factors might be associated with risk of dislocating following uncemented hemiarthroplasty (HA) due to femoral neck fracture (FNF). Current evidence is limited with great variance in reported incidence of dislocation (1–15%). Aim of this study was to identify the cumulative incidence of first-time dislocation following HA and to identify the associated risk factors.

**Method:**

We performed a retrospective cohort study of patients receiving an HA (BFX Biomet stem, posterior approach) at Copenhagen University Hospital, Bispebjerg, in 2010–2016. Patients were followed until death or end of study (dec 2018). Dislocation was identified by code extraction from the Danish National Patient Registry.

Variables included in the multivariate model were defined pre-analysis to include: age, sex and variables with a *p*-value < 0.1 in univariate analysis. A regression model was fitted for 90 days dislocation as the assumption of proportional hazard rate (HR) was not met here after.

**Results:**

We identified 772 stems (some patients occurred with both right and left hip) and 58 stems suffered 90 dislocations during the observation period, resulting in a 7% (CI 5–9) incidence of dislocation 90 days after index surgery. 55 of the 58 stems (95%) experienced the first dislocation within 90 days after surgery.

Only absence of dementia was identified as an independent protective factor in the cause-specific model (HR 0.46 (CI 0.23–0.89)) resulting in a 2.4-fold cumulative risk of experiencing a dislocation in case of dementia. Several other variables such as age, sex, various medical conditions, surgery delay and surgical experience were eliminated as statistical risk factors.

We found a decrease in survival probability for patients who experienced a dislocation during follow-up.

**Conclusions:**

The incidence of first-time dislocation of HA (BFX Biomet stem, posterior approach) in patients with a hip fracture is found to be 7% 90 days after surgery. Due to the non-existing attribution bias, we claim it to be the true incidence. Dementia was among several variables identified as the only risk factor for dislocation.

In perspective, we may consider treating patients with dementia by other methods than HA e.g., HA with cement or with a more constrained solution. Also, a surgical approach that reduce the risk of dislocation should be considered.

**Supplementary Information:**

The online version contains supplementary material available at 10.1186/s12891-024-07237-z.

## Background

Hemiarthroplasty (HA) is recommended for displaced femoral neck fracture (FNF) in elderly patients by most guidelines [1, 2]. HA is associated with risk of dislocation, and previous studies show a number of factors that affects this risk such as: cognitive function, delay in surgery, unipolar vs bipolar implant head and surgical approach (anterolateral vs. posterior) [3–6, 20]. Some of these risk factors can be explained by selection bias, and a retrospective cohort study that allows for adjustments should be performed. Such studies exist but their limitations are e.g., analysis of old implants with a unipolar head [7]. which is known to lead to an increased dislocation risk compared to the use of modern bipolar heads [8, 9].

Furthermore, there is a great variance in reported incidence of dislocation at 1% to 15% [3, 10, 11, 20]. If we truly want to compare implant performance between populations, we need to investigate the crude risk for dislocation, eliminating the influence of mortality on dislocation risk and thereby determining the unbiased incidence. Also, studies need to adjust for difference in baseline characteristics that might influence the risk of a patient experiencing dislocation of a HA. High quality studies of potential risk factors for dislocation of a HA in patients with FNF are, to the best of our knowledge, none existing for reported crude risk.

A true picture of these risk factors is needed if one wish to investigate the effect of a changed surgical technique or a new implant because the analysis must correct for them [12]. Thus, there is an urgent need to map the risk factors that influence the risk of dislocation of HA in patients with FNF, so we are able to evaluate the performance of the newest implants on the market.

The study aimed to: 1) calculate the cumulative incidence of first-time dislocation following uncemented hemiarthroplasty (uHA) in patients with FNF, and 2) identify the associated crude risk factors categorized as patient, surgeon, and implant-related.

## Methods

### Study design

The project was a retrospective cohort study of a consecutive patient population receiving an HA (BFX Biomet stem) at Copenhagen University Hospital Bispebjerg, Denmark in 2010–2016. The time period is determined by the period in which our department solely used BFX Biomet stem for HA in patients with FNF.

Patients were followed until occurrence of death or end of the study (31.12.18), whichever came first. The patients were identified by diagnostic and procedure ICD-10 codes (see Additional file 1) in our surgical planning system. Events (dislocation) were identified by diagnostic and procedure ICD-10 codes (see Additional file 2) extracted from Danish National Patient Registry (DNPR). These archives have a high validity for outcome measures for other diseases and events, but have not been validated for HA and dislocation codes [13]. Specifically, the DNPR provides nationwide longitudinal registration of detailed, survival, administrative and clinical data [13]. The DNPR extraction includes somatic contacts at both public and private hospitals in all of Denmark ensuring the completeness of the register. The DNPR enables a follow-up rate at almost 100% and thereby there were no censoring regarding inclusion of events, which it unique for this study.

To identify events (dislocation), we used a validated algorithm proposed by Hermansen et al., developed for locating dislocations of total hip arthroplasties (THA) based on codes from the DNPR [14]. Hermansen et al. found that a combination of the correct diagnoses and procedural codes increased the sensitivity from 63 to 91%. The algorithm thereby yields a sensitivity of 91% and positive predictive value of 93% and specificity greater than 99%. Even though the algorithm was developed on dislocations of THAs, we claim it to be applicable for dislocations of HAs because both patient groups were treated by the same department and personnel in our time period 2010–2018.

### Identification of cohort

Patient files were reviewed for inclusion and exclusion criteria.

The inclusion criteria were:

- 1) Patients with FNF treated with HA (BFX Biomet stem).

The exclusion criteria were:

- 1) Previously hip fracture in the same hip
- 2) Pathological fracture
- 3) Perioperatively death
- 4) Age under 50 years
- 5) Hip dysplasia diagnosis

### Variables

The baseline variables expected to be intriguing were found in multiple databases but primarily in the patient chart and to ensure data completeness some variables were supplementally identified from multiple databases (Danish Anesthesia Database (DAD) and Danish Interdisciplinary Register for Hip Fractures (RKKP hip fracture)).

#### Patient related variables

Age (years), sex (female, male), dementia (none, manifest deficiency reported in the admission record), chronic obstructive pulmonary disease (no, yes), American Society of Anesthesiologists (ASA) score (1 + 2, 3 + 4. Supplemented by DAD), Body Mass Index (BMI) (normal, obese, underweight. Supplemented by DAD and RKKP hip fracture), alcohol overuse (false, true), residence status (home, nursing home, ‘other’ which covered relief residence, rehabilitation, retirement community and group home for elderly), side of FNF (right, left. All classified as Garden stage 3–4), date of surgery, surgery delay (calculated in hours from the time between admission note with diagnostic X-ray and operation note), type of anesthesia (spinal, epidural, general anesthesia (GA). Supplemented by DAD. If more than one type of anesthesia were used (e.g., epidural and GA) it was noted as GA), date of death.

#### Surgeon related variables

Surgeon and supervisor experience (classified into ‘junior’ and ‘senior’ separated by 3 years of orthopedic surgical experience as described by Palm et al. [15]. and surgeons who advanced in training during study period was taken into account. The supervisor had to be present at the start of the operation for this expertise to count).

#### Implant related variables

Prostheses stem size and bipolar caput size. Unfortunately, these data included groups with less than 5 patients for some subgroups and therefore these variables have been discontinued due to the small test size.

### Stem design

The BFX Biomet stem is uncemented for press-fit insertion. It comes in different sizes with a collar and is made of titanium-alloy. It is fully hydroxyapatite coated surface. The bipolar head is mounted over conus with a taper -1. The stems inserted was between size 7, 9, 11, 13, 15, 17. Implant head sizes used was between 42–52 mm.

### Surgical technique

All stems were inserted by a posterior surgical approach. Whenever possible suture of the joint capsule and reinsertion of the rotators were performed by osteosuture or mainly to the soft tissue if trochanter major was used. We could not find convincing data on exactly how many operations included joint capsule suture and reinsertion of the rotators, as the standard operation description in the time period contained a description of this step and this was therefore rarely changed or omitted. In addition, some operation records could not be found and data could not be obtained from another source. Therefore, we decided not to include this as a variable.

### Statistics

Variables were considered normally distributed, thus mean and 95% confidence level are presented. Incidence was calculated by Aalen-Johansen estimator with death considered a competing event for dislocation. A subdistribution and a cause-specific Cox model was fitted to identify net and crude independent risk factors for dislocation. The results from these models were presented as hazard ratio (HR) with 95% confidence intervals (CIs). Variables included in the multivariate model was defined pre-analysis to include: age, sex, surgical experience and variables with a *p*-value < 0.1 in univariate analysis. A regression model was fitted for 90 days, as the assumption of proportional hazard rate was not met here after. Cumulative incidence function was used to identify risk of dislocation, and Grays test to identify any difference between strata. Kaplan Meier analysis was used for survival estimation and difference for survival in strata was evaluated by log-rank test.R3.2

## Results

### Participants

**Fig. 1:**
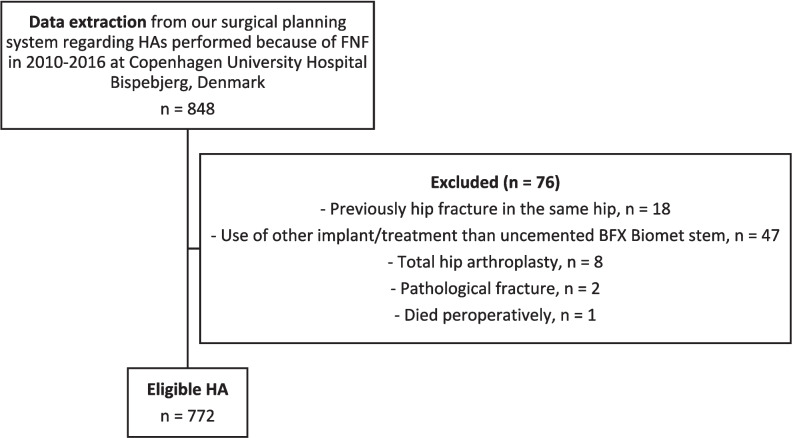
Flowchart providing an overview of data cleaning of the data extracted from our surgical planning system

The data extraction from our surgical planning system revealed that 812 patients received 848 BFX Biomet stems as treatment for a FNF in 2010–2016 at Copenhagen University Hospital Bispebjerg, Denmark. Thus 36 patients occurred twice with both right and left hip in the inclusion period, and this is why the term stem is used from now on instead of patients. In total 76 stems were excluded thus leaving 772 stems for further analysis, see Fig. 1.

### Patient demographics

In general, patients without and with a dislocation exhibited similar distributions in terms of: age, year of surgery, sex, BMI, alcohol consumption, cases of chronic obstructive pulmonary disease, ASA score, surgery delay, highest surgeon or supervisor experience and type of anesthesia.

However, patients with a dislocation were more likely to: succumb to death, suffer from manifest dementia and live with some sort of assistance. See Table 1 for full information.

**Table 1:** Demographics of all included patients with HA performed because of FNF and p-value results of univariate analysis

Variable	Complete study population *n* = 772 (no.)	HAs without dislocation *n* = 714 (mean [range]) (no. (%))	HAs with dislocation *n* = 58 (mean [range]) (no. (%))	*p*-value*
**Age (years)**	772	84 [53–105]	86 [63–100]	0.2
**Follow days (days)**	772	1,144 [0–3782]	686 [6–3217]	< 0.001
**Dead**	772	518 (73%)	49 (84%)	0.048
**Year of surgery** 2010 2011 2012 2013 2014 2015 2016	772	121 (17%) 114 (16%) 110 (15%) 106 (15%) 101 (14%) 101 (14%) 61 (8.5%)	9 (16%) 7 (12%) 10 (17%) 10 (17%) 10 (17%) 6 (10%) 6 (10%)	> 0.9
**Sex** Female Male	772	532 (75%) 182 (25%)	44 (76%) 14 (24%)	0.8
**BMI** Normal Obese Underweight	763	491 (70%) 127 (18%) 87 (12%)	40 (69%) 10 (17%) 8 (14%)	> 0.9
**Dementia** Yes No	772	211 (30%) 503 (70%)	30 (52%) 28 (48%)	< 0.001
**Alcohol overuse** False True	758	630 (90%) 72 (10%)	50 (89%) 6 (11%)	> 0.9
**Residence** Home Nursing home Other**	766	420 (59%) 189 (27%) 99 (14%)	23 (40%) 22 (38%) 13 (22%)	0.013
**Chronic obstructive pulmonary disease**	769	88 (12%)	7 (12%)	< 0.9
**ASA score** 1 + 2 3 + 4	770	302 (42%) 410 (58%)	29 (50%) 29 (50%)	0.1
**Surgery delay (hours)**	765	26 (370)	26 (313)	0.8
**Highest surgeon or supervisor experience** Junior Senior	769	220 (31%) 491 (69%)	19 (33%) 39 (67%)	0.8
**Prostheses head size**	742	(Discontinued due to small test size)		0.7
**Prostheses stem size**	757	(Discontinued due to small test size)		0.021
**Anesthesia** Epidural GA Spinal	766	130 (18%) 479 (68%) 100 (14%)	10 (18%) 41 (72%) 6 (11%)	0.7

^*^ = Wilcoxon rank sum test; Pearson's Chi-squared test; Fisher's exact test

^**^ = ‘other’ covered relief residence, rehabilitation, retirement community and group home for elderly

### Incidence

**Fig. 2:**
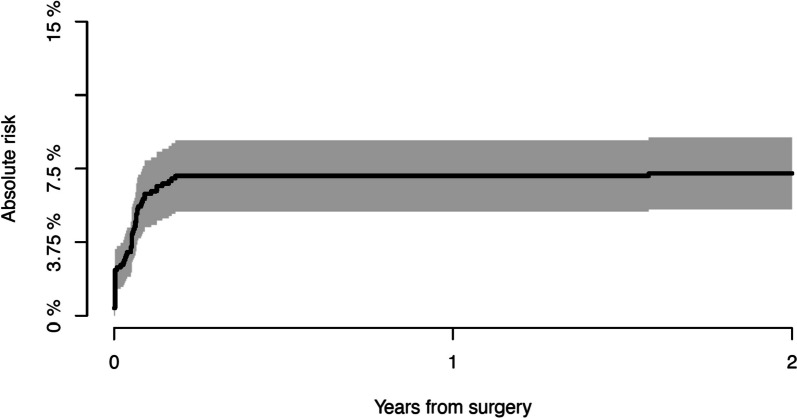
Cumulative incidence of experiencing a first-time dislocation within the first 2 years after surgery Risk factors.

We identified 90 dislocations in 58 stems during the observation period. Mean time for first-time dislocation was 61 days (range: 0–1031 days). Fifty-five of the 58 patients (95%) experienced the first-time dislocation within 90 days after surgery. This resulting in an incidence of first-time dislocation of 7% (CI: 5–9%) 90 days after surgery, and this increasing to 8% (CI: 6–9%) 8 years after surgery. See Fig. 2.

### Risk factors

A regression model was fitted for 90 days as hazard rate was not found to be constant after this point including the pre-analysis defined variables. Dementia and residence status were found as independent risk factors in subdistribution model (dementia: HR 0.46 (CI: 0.22–0.92), residence ‘other’: HR 2.04 (CI: 1.00–4.14)). Only absence of dementia was identified as an independent protective factor in the cause-specific model (HR 0.46 (CI: 0.23–0.89)). Other variables that had a *p*-value < 0.1 in univariate analysis included: surgical experience, ASA score, sex, age and stem size. But the subdistribution model showed all of these to be without association with experiencing a dislocation. See Table 2 for full cause-specific model analysis. See Additional file 3 for both full subdistribution model and cause-specific model analysis.

**Table 2:** Results of cause-specific hazard model performed with the variables that had a *p*-value < 0.1 in univariate analysis

	Cause-Specific Hazard Model		Reference
	Dislocation (HR (CIs))	Death (HR (CIs))	
Highest surgical experience	0.85 (0.48–1.51)	0.91 (0.64–1.30)	Junior
ASA score	0.63 (0.36–1.11)	2.32 (1.55–3.47)	1 + 2
Sex	1.20 (0.63–2.28)	1.51 (1.05–2.18)	Female
Residence			Home
Nursing home	1.59 (0.75–3.36)	1.71 (1.10–2.66)	
Other^a^	2.07 (0.98–4.36)	1.28 (0.78–2.10)	
Age			> 79 years
50–69 years	0.68 (0.09–5.11)	0.77 (0.31–1.91)	
70–79 years	0.82 (0.41–1.65)	0.59 (0.38–0.94)	
Dementia	**0.46 (0.23–0.89)**	0.80 (0.53–1.21)	Yes
Stem size	1.10 (0.95–1.28)	1.07 (0.98–1.17)	Continues

^a^ = relief residence, rehabilitation, retirement community, group home for elderly

**Fig. 3:**
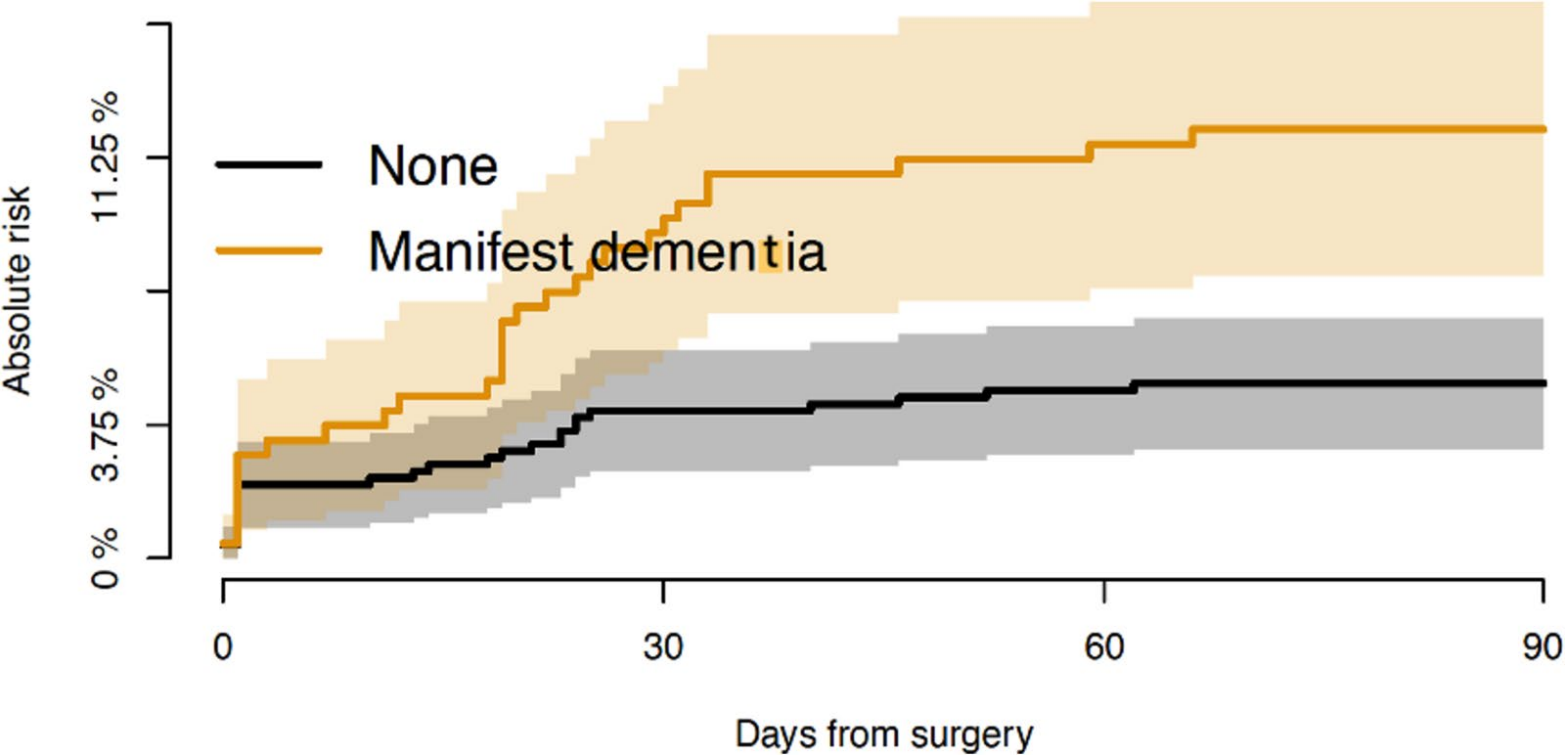
Absolute risk of dislocation for patients without and with dementia within 90 days after surgery

Patients with manifest dementia showed a 2.4-fold cumulative risk of experiencing a dislocation. The risk of experiencing a dislocation among patients with dementia was 12% (CI: 8–16%) vs only 5% (CI: 3–7%) among patients without dementia at 90 days after surgery (*p* < 0.001), see Fig. 3.

### Survival probability

**Fig. 4:**
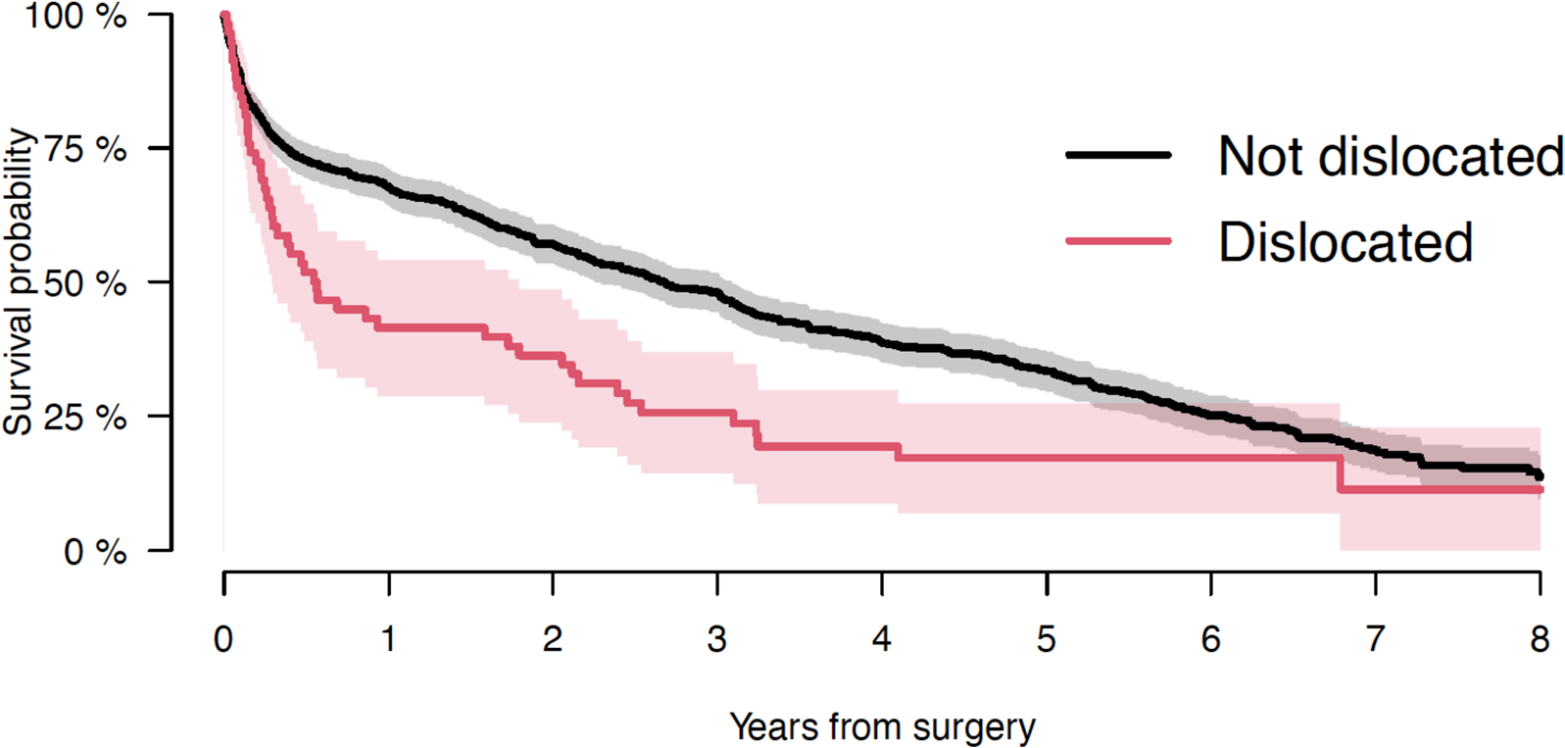
Survival probability of patients without and with dislocation of HA seen in years from surgery

Mean follow-up for patients alive at end of study was 73 months (range: 25–124), and 24 months (range: 0–101) for patients succumbed to disease during follow-up. Overall survival 1 year after surgery was 66% (CI: 62–69%). Survival 1 year after surgery for patients without and with a dislocation was respectively 68% (CI: 64–71) and 41% (CI: 29–54), (*p* < 0.001). So generally, there was a decrease in survival observed for patients who experienced a dislocation during follow-up. See Fig. 4.

## Discussion

### Incidence

In current study, we identified the cumulative incidence of first-time dislocation to be 7% (CI: 5–9%) 90 days after surgery for uncemented HA performed through a posterior approach. Our cohort comprised a complete population as the social health care system in our country eliminates bias in referral. Dislocations were identified from an algorithm by Hermansen et al. [14]. ensuring high validity from the DNPR. The DNPR contains all of our cohort except if the patient moved outside Denmark, which is extremely rare in this patient population. Due to these two aspects, we claimed our established 7% risk 90 days after surgery to be the true incidence of dislocation for patients undergoing HA with a BFX Biomet stem because of FNF performed with a posterior approach. Our findings suggested a shift in the incidence of dislocation with a cuff of at 90 days. We therefore hypothesize that causes for dislocation were different before and after the 90 days milestone. This 90 days phenomenon aligns with observations by Salem et al., who reported that 81% of dislocations in their study occurred in the first 6 weeks after surgery [16].

### Risk factors

Manifest dementia emerged as the sole prognostic risk factor for dislocation, increasing the risk 2.4 times. Falsetto et al. [20] found that presence of dementia was associated with a 1.8-fold increased risk of dislocation comparable to our findings. This elevated risk found in people with dementia may be explained by patients being less able to understand and follow a postoperative mobility regime, they move more freely and risky because they do not understand the change in joint function and they have a high risk of recurrent falls [24].

We identified death as a competing risk factor for experiencing a dislocation. This is important as present literature does not account for the high mortality observed in this patient population and they report dislocation-free survival by net failure (Kaplan Meier estimates) [17]. making comparison of implant performance difficult as survival influence the risk of dislocation in different cohorts. Moreover, some of the known risk factors for dislocation can be explained by selection bias and present studies are limited by 1) univariate analysis methods used to identify the risk factors which do not adjust for confounding [18] and 2) attrition bias [19] both factors our study is not limited by.

The most surprising variable that was not identified as a risk factor was the surgical experience, but we found no correlation between low surgical training or absence of supervisor and risk of later dislocation. This is in coherence to other studies of FNF patients [28]. Even though the supervisor had to be present at the start of the operation for this expertise to count, we had to suspect a degree of underreporting which may explain this phenomenon. Previous studies have stated that surgeries performed by unsupervised junior surgeons were an independent risk factor for reoperation of the more complex FNFs [15].

### Survival probability

We found an excess mortality in patients who experienced a dislocation of their HA compared to those without dislocation. The survival probability 1 year after surgery for patients not experiencing dislocation was 27% higher compared to patients experiencing dislocation. Due to the non-existing attrition bias because of thorough survival registries in our country, the validity of competing risk and survival analysis in this current study was unique. The decrease in survival may be explained by the fact that a dislocation triggers an admission and thus the risk of a nosocomial infection or the associated anaesthesia may affect the patient’s general health. Another explanation might by that the dislocation results in a temporary impairment of function. Falsetto et al. has similarly observed this trend and they explained it by the fragile patient group [20]. We advocate that in future studies a greater awareness of dislocations association to change in survival is in scope.

### Limitations and strengths

An overall limitation of this study was underreporting. Even though we tried to complete the data of baseline variables by searching multiple databases and supplementing them with each other some data was just not recorded thus missing in the multivariate analysis. This may lead to underpower in analysis.

There was a potential underreporting of dislocation ICD-10 coding because a ‘closed reposition of dislocation’ code does not exist and because closed repositions could be performed in emergency departments without a hospital admission [21]. However, we feel confident that the validity of the DNPR coding shown for total hip replacement [14]. ensures a positive predictive value of 96.6% and a negative predictive value of 99.8% for our cohort as well and thereby limiting this reporting bias.

Another limitation was the opt-out of radiographic findings and thereby component positioning as this could have influenced the result as shown for total hip replacement surgery [21].

Regarding the study design the retrospective non-randomized design of this study limited the strength of evidence of our findings. But the population-based design was a strength, as no loss to inclusion limited our study and the results were thus applicable for a general Scandinavian population.

We exclusively used a posterior approach for stem insertion. Studies have shown this choice to increase the risk of dislocation compared to the direct anterior, anterolateral and the newer SPAIRE approach. The most common used approaches are the anterolateral and posterior [3]. where the anterolateral approach has a reported incidence of dislocation between 0 and 3,3% [3]. The direct anterior approach has an incidence between 0 and 2% [26]. The newer ‘Saving Piriformis And Internus, Repair of Externus’ approach is a muscle sparing mini-posterior approach and it has an incidence at 0,3% [27]. With this variance in dislocation incidence in mind our findings were only relatable to a population of patients undergoing the posterior surgical approach. However, in most cases in our cohort the operations included suture of the joint capsule and reinsertion of the rotators, which increases the stability of the prosthesis despite the posterior approach.

Also, as we only included a single stem (BFX Biomet) our results may not be valid for other stems. We did, however, provide the cumulative incidence of dislocation making our results a reliable reference for comparison of dislocation risk between stems in unrelated cohorts which is an advance and strength in this current study.

### Perspectivation

In a clinical context, we wanted to use our findings to optimize the risk of experiencing a dislocation of a HA in patients with a FNF, and since dementia was the only risk factor, our focus lay here. Since patients with dementia often have been excluded from previous trials and the ageing population contains an increasing number of patients with dementia, it was of great importance to look at this patient group [22, 23]. In perspective, we may consider to treat patients with dementia by other methods than HA e.g., HA with cement to allow for optimal stem insertion based on a trial reduction, or with a more constrained solution such as dual mobility cup [24, 25]. Also, another surgical approach should be considered, as the posterior approach is known to massively increase the risk of dislocation [5].

## Conclusions

The cumulative incidence of first-time dislocation of HA (BFX Biomet stem, posterior surgical approach) in patients with FNF is found to be 7% 90 days after surgery. Dementia is among several variables identified as the sole risk factor, and death is established as a competing risk factor for dislocation. We found an excess mortality in patients who experienced a dislocation of their HA compared to those without dislocation. We advocate that in future studies a greater awareness of dislocations association to change in survival is in scope.

In perspective, we may consider to treat patients with dementia by other methods than HA e.g., HA with cement or with a more constrained solution such as dual mobility cup. Also, another surgical approach should be considered, as the posterior approach is known to massively increase the risk of dislocation.

### Supplementary Information

**Additional file 1.**

**Additional file 2.**

**Additional file 3.**

## Data Availability

The datasets used and analyzed during the current study are available from the corresponding author on reasonable request.

## Abbreviations

ASA score: American society of anesthesiologists’ score
BMI: Body mass index
CI: Confidence interval
DAD: Danish Anesthesia Database
DNPR: Danish National Patient Registry
FNF: Femoral neck fracture
GA: General anesthesia
HA: Hemiarthroplasty
HR: Hazard ratio
RKKP hip fracture: Danish Interdisciplinary Register for Hip Fractures
THA: Total hip arthroplasties
uHA: Uncemented hemiarthroplasty
