# Supplementary material for: Incidence of dislocation and associated risk factors in patients with a femoral neck fracture operated with an uncemented hemiarthroplasty
Source: BMC Musculoskelet Disord. 2024 Feb 9;25:119. doi: 10.1186/s12891-024-07237-z (PMC10854108; doi:10.1186/s12891-024-07237-z)
Supplement: Supplementary file 1 — Additional file 1. [file 12891_2024_7237_MOESM1_ESM.docx]

Additional file 1

The patients in this study were identified by the following diagnostic and procedure ICD-10 codes in our surgical planning system:

| **Code** | **Description** |
| --- | --- |
| DS72.0 | Fracture of femoral neck |
| DS72.1 | Pertrochanteric femoral fracture |
| DS72.1A | Intertrochanteric femoral fracture |
| DS72.1B | Trochanteric femoral fracture |
| DS72.2 | Subtrochanteric femoral fracture |
| DS72.7 | Multiple femoral fracture |
| DS72.8 | Fracture of another part of femur |
| DS72.8A | Fracture of femoral head |
| DS72.9 | Fracture of femur |
| KNFB02 | Hemiarthroplasty |
| KNBB12 | Hemiarthroplasty with cement |
