# Supplementary material for: Incidence of dislocation and associated risk factors in patients with a femoral neck fracture operated with an uncemented hemiarthroplasty
Source: BMC Musculoskelet Disord. 2024 Feb 9;25:119. doi: 10.1186/s12891-024-07237-z (PMC10854108; doi:10.1186/s12891-024-07237-z)
Supplement: Supplementary file 2 — Additional file 2. [file 12891_2024_7237_MOESM2_ESM.docx]

Additional file 2

Events (dislocation) in this study were identified by the following ICD-10 codes extracted from DNPR (Danish National Patient Registry):

| **Code** | **Description** |
| --- | --- |
| DS72.0 | Fracture of femoral neck |
| DS72.1 | Pertrochanteric femoral fracture |
| DS72.1A | Intertrochanteric femoral fracture |
| DS72.1B | Trochanteric femoral fracture |
| DS72.2 | Subtrochanteric femoral fracture |
| DS72.7 | Multiple femoral fracture |
| DS72.8 | Fracture of another part of femur |
| DS72.8A | Fracture of femoral head |
| DS72.9 | Fracture of femur |
| DS73.0 | Dislocation of hip |
| DT84.0* | Mechanical complication of joint prosthesis |
| DT84.3 | Mechanical complications of another bone prosthesis, implant or graft |
| DT84.4* | Mechanical complication of orthopedic prosthesis, implant or graft |
| DT84.8 | Another complication of orthopedic prosthesis, implant or graft |
| DT84.9 | Another complication of orthopedic prosthesis, implant or graft (without further specification) |
| DZ98.8 | Another postoperative condition |
| KNF* | All surgery in the hip region |
| KNBB12 | Hemiarthroplasty with cement |
| KNDH12 | Open reposition of dislocation |

(* = including any subdivision)
