# Supplementary material for: Incidence of dislocation and associated risk factors in patients with a femoral neck fracture operated with an uncemented hemiarthroplasty
Source: BMC Musculoskelet Disord. 2024 Feb 9;25:119. doi: 10.1186/s12891-024-07237-z (PMC10854108; doi:10.1186/s12891-024-07237-z)
Supplement: Supplementary file 3 — Additional file 3. [file 12891_2024_7237_MOESM3_ESM.docx]

Additional file 3

Results of both subdistribution hazard model and cause-specific hazard model performed with the variables that had a p-value <0.1 in univariate analysis. Only dementia showed an association with dislocation in the final cause-specific hazard model.

|  | Subdistribution Hazard Model | | Cause-Specific Hazard Model | | Reference |
| --- | --- | --- | --- | --- | --- |
|  | Dislocation | Death | Dislocation | Death |  |
| Highest surgical experience | 0.86 (0.48-1.51) | 0.93 (0.65-1.33) | 0.85 (0.48-1.51) | 0.91 (0.64-1.30) | Junior |
| ASA score | 0.61 (0.35-1.09) | 2.41 (1.59-3.64) | 0.63 (0.36-1.11) | 2.32 (1.55-3.47) | 1 + 2 |
| Sex | 1.16 (0.62-2.17) | 1.46 (1.00 - 2.13) | 1.20 (0.63-2.28) | 1.51 (1.05-2.18) | Female |
| Residence |  |  |  |  | Home |
| Nursing home | **1.46 (0.69-3.11)** | 1.67 (1.09-2.26) | **1.59 (0.75-3.36)** | 1.71 (1.10-2.66) |  |
| Other* | **2.04 (1.00-4.14)** | 1.24 (0.76-2.02) | **2.07 (0.98-4.36)** | 1.28 (0.78-2.10) |  |
| Age |  |  |  |  | > 79 years |
| 50-69 years | 0.73 (0.10-5.40) | 0.76 (0.31-1.85) | 0.68 (0.09-5.11) | 0.77 (0.31-1.91) |  |
| 70-79 years | 0.87 (0.43 -1.73) | 0.61 (0.39-0.95) | 0.82 (0.41-1.65) | 0.59 (0.38-0.94) |  |
| Dementia | **0.46 (0.22-0.92)** | 0.84 (0.57-1.24) | **0.46 (0.23-0.89)** | 0.80 (0.53-1.21) | Yes |
| Stem size | 1.10 (0.93-1.30) | 1.07 (0.97-1.17) | 1.10 (0.95-1.28) | 1.07 (0.98-1.17) | Continues |

* = relief residence, rehabilitation, retirement community, group home for elderly
